# Influence of intramolecular hydrogen bonds on the binding potential of methylated β-cyclodextrin derivatives

**DOI:** 10.3762/bjoc.8.218

**Published:** 2012-11-06

**Authors:** Gerhard Wenz

**Affiliations:** 1 Organic Macromolecular Chemistry, Saarland University, Campus Saarbrücken C4.2, 66123 Saarbrücken, Germany

**Keywords:** binding constant, cyclodextrin, hydrogen bond, methylation, regioselective

## Abstract

Various heptasubstituted derivatives of β-cyclodextrin (β-CD) bearing 1, 2 and 3 methyl substituents per glucose unit were synthesized by regioselective methods. Binding free energies and binding enthalpies of these hosts towards 4-*tert*-butylbenzoate and adamantane-1-carboxylate were determined by isothermal titration microcalorimetry (ITC). It was found that methyl substituents at the secondary positions of β-CD lead to a tremendous reduction of the binding potential, while methylation at the primary positions significantly improved binding. Stabilizing intramolecular hydrogen bonds between the glucose units were made responsible for the high binding potentials of those β-CD derivatives that possess secondary hydroxy groups.

## Introduction

Cyclodextrins (CDs) are a well-known class of organic hosts able to include various guests, preferably in aqueous solution [1–3]. Inclusion is mainly driven by hydrophobic and van der Waals interactions [4–6]. The host–guest complexes, so-called cyclodextrin inclusion compounds, find many applications such as solubilization of pharmaceutical drugs, dispersion of cosmetics, catalysis, or chromatographic separation of enantiomers [2,7–8]. Application of β-CD **1** is hampered by its low solubility of 18.8 g L^−1^ at 25 °C [9]. Solubility of β-CD and its inclusion compounds can be significantly increased by the covalent attachment of neutral or ionic substituents [10]. Methylated β-CDs, such as heptakis(2,6-di-*O*-methyl)-β-CD **2** and heptakis (2,3,6-tri-*O*-methyl)-β-CD **3**, are well known for their high solubilities in water (**2**: > 300 g L^−1^) and their interesting inclusion behavior [11–13]. Because of the tedious synthesis of the disubstituted derivative **2** [11,14], the readily available randomly substituted derivative RAMEB with a degree of substitution DS = 1.7–1.8 is preferred nowadays and produced on an industrial scale [15].

Methylated CDs have already found several applications in drug delivery [10] and polymer chemistry [16]. They allow radical polymerizations of hydrophobic vinyl monomers in homogenous aqueous solution [17–20] and living RAFT polymerizations as well [21]. Methylated CDs are already applied industrially on a large scale, e.g., for switching the viscosity of polymeric thickeners [22], for decontamination of soil [23–24], or for cosmetic formulations [25]. High binding potentials of the methylated CDs are essential for their specific functions in these applications. Therefore, a quantitative understanding of the binding potential as a function of the degree and pattern of methylation is highly desirable.

The attachment of methyl groups to β-CD improves its solubility in water because it reduces formation of intermolecular hydrogen bonds. Methylation should also extend the hydrophobic cavity of β-CD and therefore improve the binding potential for hydrophobic guest molecules. Up to now, only little is known about the influence of methyl substituents on the inclusion potential of β-CD [26–27]. The dimethyl derivative **2** binds adamantane derivatives with a similar binding constants *K* to those of native β-CD, while the trimethyl derivative **3** binds much more weakly [28]. Similar differences in binding affinities between native **1** and permethylated β-CD **3** were observed for the inclusion of anti-inflammatory drugs [29].

**Figure 1:**
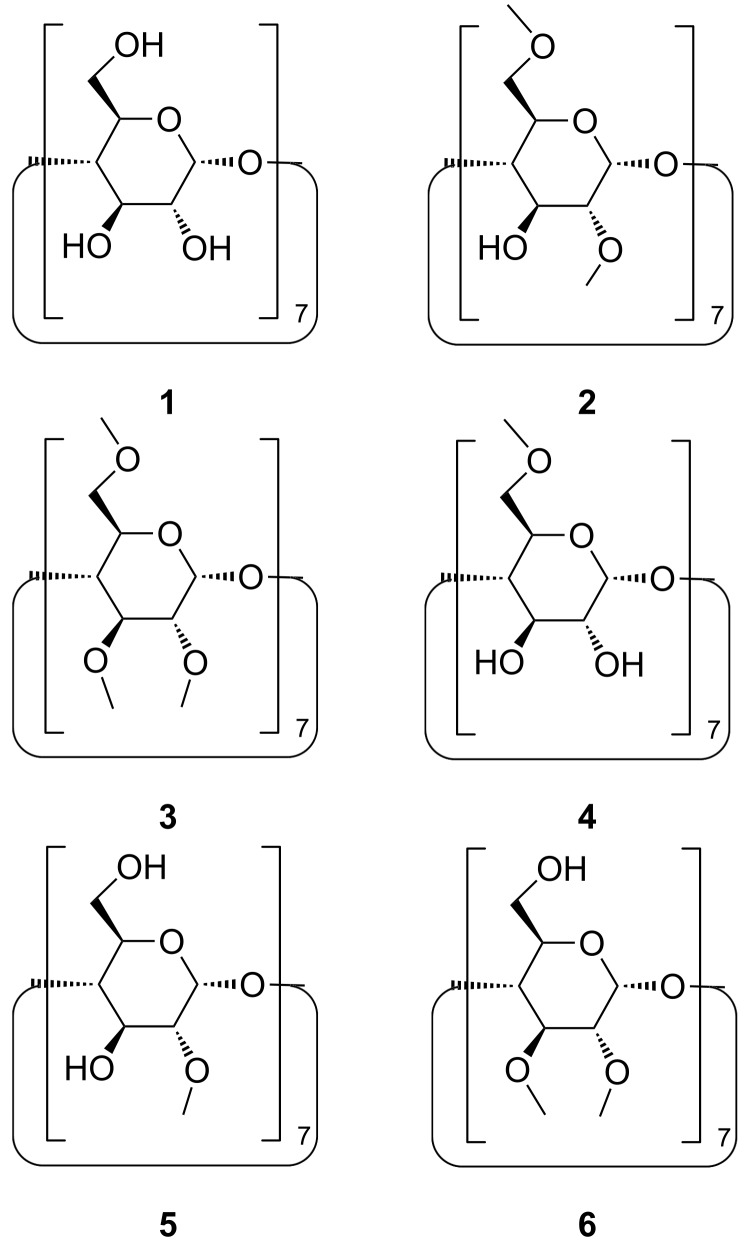
Structures of the methylated β-CD derivatives investigated.

For the systematic investigation of the influence of the pattern of methylation on the complexation of amphiphilic guests, we synthesized well-defined model compounds **2**–**6** (Figure 1) of methylated β-CD, using regioselective procedures already published [30]. 4-*tert*-Butylbenzoate and adamantane-1-carboxylate were chosen as representative guests. Complexation of these guests should sensitively respond to changes in the methylation pattern, because they fit tightly into the cavity of β-CD giving rise to high binding constants [26–27]. Binding data were measured by isothermal titration calorimetry (ITC) because it is known to be the most accurate method, and because it additionally yields binding enthalpies and entropies [31–32].

## Results

Since methylated β-CD derivatives **2**–**6** are highly water-soluble they are well suited for ITC. The ITC titration curves for all the β-CD derivatives **1**–**6** were exothermic and were in accordance with a 1:1 stoichiometry of the host–guest complexes. Thermodynamic data obtained for the guests 4-*tert*-butylbenzoate and adamantane-1-carboxylate are listed in Table 1 and Table 2, respectively.

**Table 1:** Thermodynamics of the inclusion of 4-*tert*-butyl-benzoate in β-cyclodextrin **1** and its methyl derivatives **2**–**6**.

Host	No.	*K* (M^−1^)	Δ*G*° (kJ mol^−1^)	Δ*H*° (kJ mol^−1^)	−*T*Δ*S*° (kJ mol^−1^)

unsubstituted β-CD	**1**	16400 ± 4	−24.34	−19.00 ± 0.08	−3.82
2,6-di-*O*-methyl-β-CD	**2**	17000 ± 485	−24.13	−19.98 ± 0.14	−4.18
2,3,6-tri-*O*-methyl-β-CD	**3**	1190 ± 21	−17.54	−30.54 ± 0.37	12.98
6-*O*-methyl-β-CD	**4**	30700 ± 898	−25.60	−20.14 ± 0.12	−5.49
2-*O*-methyl-β-CD	**5**	12300 ± 428	−23.33	−14.30 ± 0.11	−9.05
2,3-di-*O*-methyl-β-CD	**6**	869 ± 28	−16.77	−19.24 ± 0.84	+2.45
RAMEB^a^	**7**	14700 ± 363	−23.77	−14.60 ± 0.09	−9.20

^a^randomly methylated β-CDs.

**Table 2:** Thermodynamics of the inclusion of adamantane-1-carboxylate in β-cyclodextrin **1** and its methyl derivatives **2**–**6**.

Host	No.	*K* (M^−1^)	Δ*G*° (kJ mol^−1^)	Δ*H*° (kJ mol^−1^)	*−T*Δ*S°* (kJ mol^−1^)

unsubstituted β-CD	**1**	38100 ± 1150	−26.13	−22.38 ± 0.09	−3.78
2,6-di-*O*-methyl-β-CD	**2**	20400 ± 975	−24.58	−20.75 ± 0.22	−3.87
2,3,6-tri-*O*-methyl-β-CD	**3**	606 ± 43	−15.87	−19.94 ± 0.82	+4.04
6-*O*-methyl-β-CD	**4**	56400 ± 2400	−27.10	−19.11 ± 0.15	−8.02
2-*O*-methyl-β-CD	**5**	18700 ± 275	−24.37	−20.85 ± 0.05	−3.57
2,3-di-*O*-methyl-β-CD	**6**	586 ± 65	−15.79	−12.72 ± 0.70	−3.09
RAMEB^a^	**7**	15300 ± 341	−23.87	−15.48 ± 0.09	−8.41

^a^randomly methylated β-CDs.

Remarkable differences in the binding constants for 4-*tert*-butylbenzoate were found for the β-CD derivatives **2**–**7**. The completely methylated β-CD **3** and the 2,3-dimethylated derivative **6** showed the lowest binding constants *K*, less than one tenth of the one of native β-CD **1**. These very low binding constants are accompanied by positive values of the entropy term −*T*Δ*S°* weakening the binding free enthalpy Δ*G*°. On the other hand, binding constants of the 2,6-di-*O*-methyl derivative **2** as well as the 6-*O*-methyl derivative were higher than the one of β-CD. Apparently, methylations of secondary hydroxy groups lead to a decrease of the binding constant, while methylation at primary hydroxy groups leads to an increase. For the 2,6-di-*O*-methyl derivative both effects seem to compensate each other giving rise to a binding constant *K* similar to the one of native β-CD. The randomly methylated β-CD **7** also showed an inclusion potential very similar to β-CD for the same reason.

**Figure 2:**
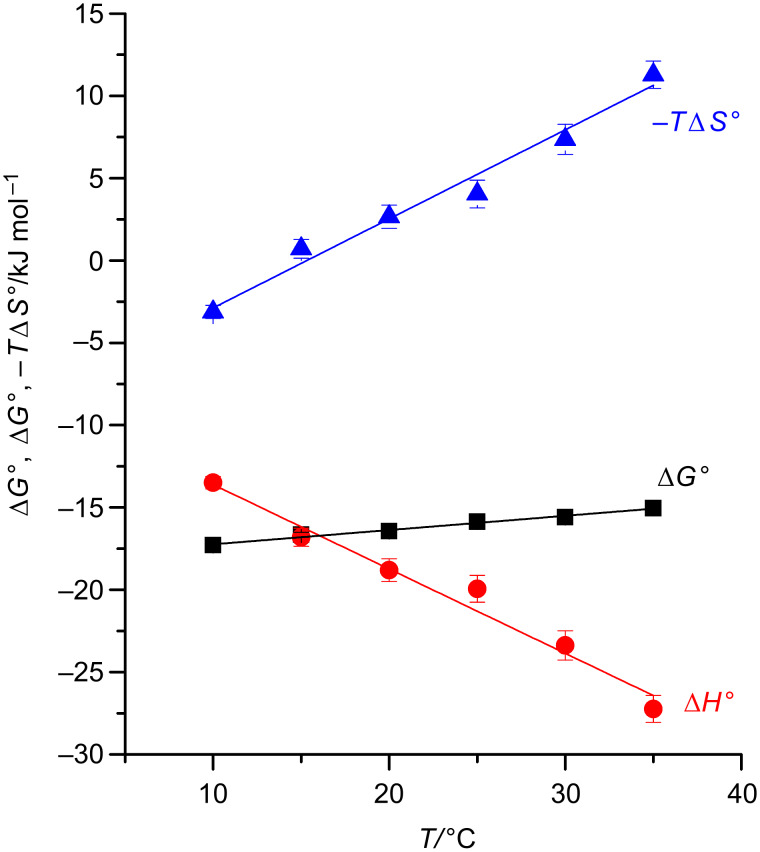
Temperature dependence of −*T*Δ*S°*, Δ*H°* and Δ*G°* for the inclusion of 1-adamantane carboxylate in heptakis-2,3,6-tri-*O*-methyl-β-CD (**3**) measured by ITC*.*

The thermodynamic data (Table 2) measured for the inclusion of adamantane-1-carboxylate in β-CD and β-CD derivatives **2**–**6**, showed a similar trend to that observed before. This guest, which is known as one of the most suitable guests for the β-CD cavity, was bound even more weakly by the 2,6-di-*O*-methyl derivative **2** than by native β-CD **1**. Again, all β-CD derivatives methylated at the secondary positions showed much lower affinities towards this guest than **1** did. Again, a positive value of the entropy term −*T*Δ*S°* was found for 2,3,5-tri-*O*-methyl-β-CD. As shown in Figure 2, this entropy term further grew with increasing temperature, compensating most of the strongly negative binding enthalpy Δ*H°.* Taking into account these data and previous results from literature, the observed reduction of the binding potential by substitutions at the secondary positions appeared to be a general feature of β-CD.

In addition, the differential heat capacity, Δ*C*_p_ = −510 ± 30 J mol^−1^ K^−1^, was calculated from the slope of the temperature dependence of Δ*H*°. Negative Δ*C*_p_ are generally interpreted as the liberation of “hot” water molecules during complexation of the guest [33–35]. The liberation of water molecules of high energy from a cavity is regarded as a major driving force for the complexation of neutral guests by concave hosts in water, because it can lead both to entropy gains and enthalpic advantages [36]. The observed value for 2,3,5-tri-*O*-methyl-β-CD is even higher than that for native β-CD, Δ*C*_p_ = −320 ± 20 J mol^−1^ K^−1^ [37]. This difference was attributed to the larger internal hydrophobic surface of 2,3,5-tri-*O*-methyl-β-CD compared to native β-CD leading to the liberation of more bound water molecules during complexation. Nevertheless, the effect of the negative heat capacity on binding adamantane carboxylate by 2,3,5-tri-*O*-methyl-β-CD is overcompensated by a strong increase of binding entropy leading in total to a reduction of the complex stability with increasing temperature.

## Discussion

Only methylations at the primary positions lead to the anticipated increase of the binding potential of β-CD due to an elongation of the hydrophobic cavity of β-CD. The negative effect of methylations at the secondary positions was initially surprising. The discussion of the entropy term −*T*Δ*S°* appeared most appropriate to us to understand this behavior.

**Figure 3:**
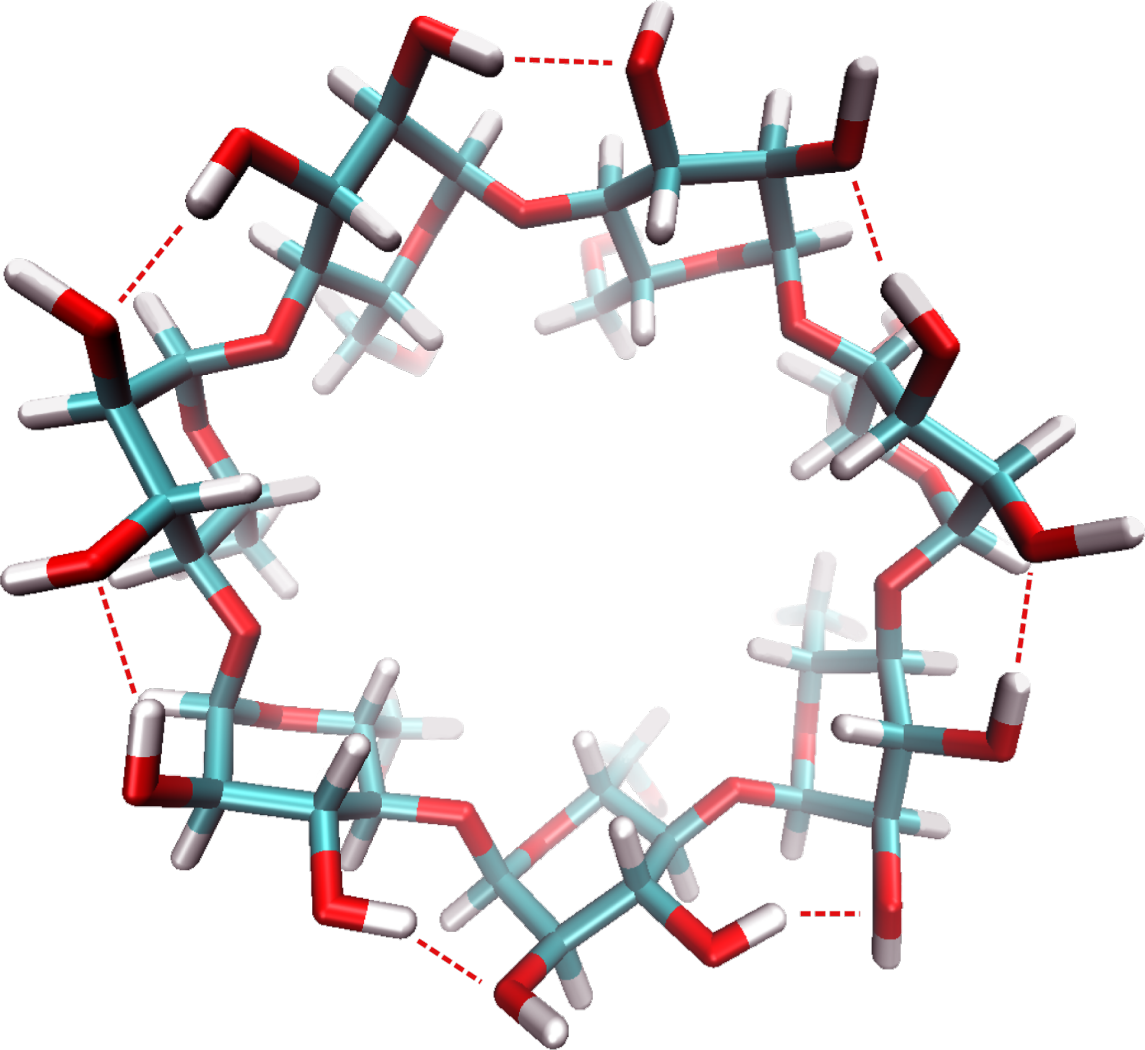
Structural drawing of β-CD [43], according to structural data (CSD-ID BUVSEQ03) from Zabel et al. [38], generated by VMD 1.8.4 showing the intramolecular hydrogen bonds between OH-3 and O-2.

The entropy term −*T*Δ*S°* was negative (−3 to −9 kJ mol^−1^) for those β-CD derivatives (**1**,**2**,**4**,**5** and **7**) equipped with free secondary hydroxy groups. This negative value is quite normal and attributed to the liberation of bound water molecules from the cavity, while the entropy of the host remains more or less unchanged [5–6]. Neutron scattering studies revealed that native β-CD is strongly rigidified by intermolecular hydrogen bonds (flip-flop bonds) between the secondary hydroxy groups of adjacent anhydroglucose units, as depicted in Figure 3 [38]. This finding was confirmed by MD calculations of CDs in the crystalline state and in aqueous environment [39–40]. A stabilization energy due to all O_3_H∙∙∙∙O_2_’ hydrogen bonds of 14 to 23 kJ mol^−1^ was calculated by using density functional theory (basis set B3LYP) [41]. In addition, recent density-functional calculations also took into account strong intermolecular hydrogen bonds of these hydroxy groups with water molecules in aqueous solution [42].

The conformational stabilization of β-CD by these hydrogen bonds between the secondary hydroxy groups is lost upon methylation. Furthermore, methylated β-CDs are less hydrated than the native ones [44]. The lack of stabilizing hydrogen bonds leads to much higher ring flexibilities for derivatives **3** and **6**, which explains the low or even positive entropy contributions −*T*Δ*S°* to the binding free enthalpy Δ*G°*. Especially for a guest such as adamantane-1-carboxylate, which fits well into the CD cavity, its inclusion will significantly reduce the conformational degrees of freedom of a flexible host, such as **3** or **6**, leading to an unfavorable decrease in entropy.

In contrast, primary hydroxy groups in β-CD are too far apart from each other to allow intramolecular hydrogen-bond formation. Hydrogen bonds between primary hydroxy groups were only found for α-CD, leading to a conical host conformation, which is unfavorable for the accommodation of a guest [45]. Therefore, methylation at the primary positions should not significantly diminish the rigidity of the CD torus. This explains why methylation at the primary position leads to the expected improvement of the binding potential. Also the substitution of the primary hydroxy groups with other hydrophobic groups, such as thioether moieties, is known to furnish host molecules with much higher binding potentials than native β-CD [46–47].

## Conclusion

The binding potential of β-CD can be improved significantly if hydrophobic substituents are exclusively attached at the primary positions. Intramolecular hydrogen bonds between secondary hydroxy groups of β-CD are crucial for achieving high binding constants. This result provides an example of a host, where intramolecular hydrogen bonds control the binding potential [48]. It will help to better understand binding mechanisms in supramolecular systems [48] and in the future help to design improved hosts based on β-CD for specific applications such as drug delivery [49], removal of pollutants [50–53], catalysis [54] or smart materials [55].

## Experimental

4-*tert*-Butylbenzoic acid and adamantane-1-carboxylic acid and β-CD derivative **3** were purchased from Aldrich, β-CD **1** and RAMEB **7** from Wacker Chemie, β-CD derivative **2** from Cyclolab. β-CD derivatives **4**–**6** were synthesized from β-CD **1** following published procedures (Table 3) [30].

**Table 3:** List of the methylated β-CD derivatives used in this contribution.

Host	No.	CAS Registry No.	Reference

unsubstituted β-CD	**1**	7585-39-9	
2,6-di-*O*-methyl-β-CD	**2**	51166-71-3	[11]
2,3,5-tri-*O*-methyl-β-CD	**3**	55216-11-0	[11]
6-*O*-methyl-β-CD	**4**	84337-62-2	[30]
2-*O*-methyl-β-CD	**5**	60786-23-4	[30]
2,3-di-*O*-methyl-β-CD	**6**	123155-05-5	[30]
RAMEB^a^	**7**	343249-39-8	[15]

^a^randomly methylated β-CDs.

Binding data were measured with isothermal microcalorimetric titration at a temperature of 25.0 °C with an AutoITC isothermal titration calorimeter (MicroCal Inc., Northampton, USA) by using 1.4144 mL sample and reference cells. The reference cell was filled with distilled water. The sample cell was filled with a 2 mM solution of the respective host in 50 mM phosphate buffer pH 6.8, and the contents were constantly stirred at 450 rpm. A 26 mM solution of the guest was prepared in the same buffer and adjusted by the addition of small quantities of HCl or NaOH to pH 6.8. This solution was automatically added by a syringe in 20 separate injections of 12.5 µL. The resulting 20 heat signals were integrated to yield the mixing heats, which were corrected by the corresponding dilution enthalpies of β-CD in the same buffer. The titration curve was fitted by nonlinear regression using the program Origin 7.0 for ITC. Thereby, a 1:1 stoichiometry of the guest and the host molecule was found to be most appropriate. The binding constant *K* and the molar binding enthalpy *∆H°* were obtained as fitting parameters, from which the binding free energy *∆G°* and binding entropy *∆S°* were derived. For those titrations with high binding constants, i.e., *K* > 5000 M^−1^, titrations were repeated with [host] = 10/*K* in the cell and [guest] = 13[host] in the syringe to ensure optimal accuracy of the nonlinear-regression fitting procedure.
